# Contemporary Review of Clinical Features, Multi-Modality Imaging, and Management of Pericardial Cysts

**DOI:** 10.3390/jcm15041585

**Published:** 2026-02-18

**Authors:** Ankit Agrawal, Mohab Elnashar, Keshav Garg, Ahmad Mustafa, Akiva Rosenzveig, Aro Daniela Arockiam, Elio Haroun, Rishabh Khurana, Allan L. Klein, Tom Kai Ming Wang

**Affiliations:** 1 Department of Cardiovascular Medicine, University of Arkansas for Medical Sciences, Little Rock, AR 72211, USA; 2 Center for the Diagnosis and Treatment of Pericardial Diseases, Section of Cardiovascular Imaging, Department of Cardiovascular Medicine, Heart, Vascular and Thoracic Institute, 9500 Euclid Avenue, Main Campus J1-5, Cleveland Clinic, Cleveland, OH 44195, USA; 3 Department of Internal Medicine, Unity Health, Searcy, AR 72143, USA; 4 Department of Internal Medicine, Yale New Haven Health System, New Haven, CT 06510, USA; 5 Section of Cardiovascular Imaging, Imaging Institute, Cleveland Clinic, Cleveland, OH 44195, USA

**Keywords:** pericardial cysts, pericarditis, pericardium

## Abstract

Pericardial cysts (PCs) are rare, benign congenital abnormalities that are encountered as mediastinal lesions. Despite their rarity, they remain clinically important due to their potential to mimic other mediastinal or cardiac pathologies and their capacity, in select cases, to cause significant complications. PCs are typically identified incidentally on imaging studies such as chest x-ray or transthoracic echocardiography, as most patients remain asymptomatic throughout their lives. When symptoms do occur, they are often nonspecific and related to compression of adjacent structures. Serious complications—including infection, rupture, and, rarely, cardiac tamponade—have been reported, underscoring the importance of accurate diagnosis and appropriate follow-up. Definitive characterization of PCs is best achieved using advanced imaging modalities such as cardiac computed tomography or cardiac magnetic resonance imaging, which help differentiate PCs from other mediastinal masses. While many PCs remain stable or even regress spontaneously, intervention may be warranted for symptomatic patients, enlarging cysts, or when the diagnosis remains uncertain. Therapeutic options include percutaneous aspiration, which carries a risk of recurrence, and surgical resection, which offers definitive treatment with excellent outcomes. This review provides a comprehensive overview of the etiology, clinical manifestations, diagnostic evaluation, differential diagnosis, complications, and management strategies for PCs.

## 1. Introduction

Pericardial cysts (PCs) are rare, benign, fluid-filled lesions of the mediastinum, accounting for less than 6% of all mediastinal masses and occurring in approximately 1 in 100,000 individuals [1,2]. Most PCs are congenital malformations, although acquired forms have been described, particularly following cardiothoracic surgery or infection [3,4]. With the widespread use of advanced imaging modalities such as chest computed tomography (CT), cardiac magnetic resonance imaging (CMR), and echocardiography, the incidental detection of PCs has increased substantially [3,5,6].

Although the majority of PCs are asymptomatic and discovered incidentally, their clinical presentation can vary widely depending on size, location, and relationship to adjacent cardiovascular or respiratory structures. When symptomatic, PCs may cause chest pain, dyspnea, cough, arrhythmias, or, rarely, life-threatening complications such as cardiac compression, tamponade, or infection. Furthermore, PCs can mimic other mediastinal or cardiac masses, posing diagnostic challenges and raising concerns regarding malignancy or the need for intervention. As a result, accurate imaging-based characterization is essential to establish the diagnosis, assess potential complications, and guide management decisions.

PCs are uncommon but clinically relevant entities that require a nuanced diagnostic and management approach. Improved understanding of their natural history and evolving evidence-based surveillance strategies can help guide optimal patient care. This review aims to discuss the clinical features, evaluation, and management of PCs.

## 2. Methodology

Our article is a contemporary review of the literature focusing on the epidemiology, clinical presentation, imaging evaluation, and management of pericardial cysts. A literature search was performed using PubMed to identify relevant articles published in English from database inception through 2025. Search terms included combinations of “pericardial cyst,” “mediastinal cyst,” “pericardial mass,” “cardiac cyst,” and “pericardial lesions.” Additional relevant studies were identified by manual review of reference lists from key articles and review papers. The selection of articles was guided by clinical relevance and contribution to understanding the diagnosis, imaging characteristics, natural history, and management of pericardial cysts. Review articles, observational studies, case series, and illustrative case reports were included. Studies in non-English languages, or focusing on non-pericardial mediastinal cysts, non-cardiac pathology, or lacking clinical relevance were excluded. Given the narrative nature of this review, no formal study quality assessment or quantitative synthesis was performed.

## 3. Pathophysiology and Etiology

PCs can be simple or complex cysts. Simple PCs are thin-walled, unilocular, fluid-filled lesions with homogeneous internal content, while complex PCs may exhibit features such as thick or irregular walls, septations, internal debris, or heterogeneous content [3]. Simple cysts are more prevalent and usually measure < 5 cm, although a size range of 2–28 cm has been described [1,7]. PCs can be congenital or acquired, with the former being more common [8,9]. The pericardium develops as a distinct mesothelial sac derived from progenitor cells migrating from the lateral plate mesoderm. This migratory process occurs independently of cardiac morphogenesis within the heart fields [10]. Disruption of this migration can lead to incomplete coalescence of the mesenchymal lacunae that form the pericardial sac. A noncommunicating lacuna gives rise to a fluid-filled monocular cavity lined by mesothelial cells, known as a congenital PCs [3]. Less commonly, PCs can occur due to surgical trauma disrupting pericardial layers, creating a potential space lined by mesothelial cells. Post-inflammatory cysts occur after a similar disruption occurs, which constitute the acquired cysts, like in rheumatic pericarditis, post-myocardial infarction pericarditis, latent viral infectious pericarditis, and post-traumatic or post-cardiac surgery pericarditis [7,11,12]. Hydatid cysts are a rare cause of PCs [3,4,13].

## 4. Epidemiology and Clinical Features

PCs represent 7% of all masses found in the mediastinum and constitute 33% of mediastinal cysts [14]. Although they can appear in multiple locations, they are predominantly found within cardiophrenic angles. Their most frequent site is the right cardiophrenic angle, seen in 51–70% of instances, with the left cardiophrenic angle being the second most common (22–38%) [15]. In a smaller subset, from 8% to 11% of patients, these cysts are located in atypical areas, including the posterior mediastinum, hilar regions, the paratracheal space, or near the aortic arch [16]. Most cysts are unilocular, measure between 2 and 4 cm in diameter, and are usually detected incidentally in individuals aged between 30 and 50 years [17]. Typically, PCs are discovered in adults between the third and fourth decades of life, most often as asymptomatic findings adjacent to the right cardiophrenic angle on chest radiography [3].

While the majority of cases remain clinically silent, up to 25% of patients may present with nonspecific symptoms, including chest pain, cough, dyspnea, or palpitations, and rare complications such as cardiac tamponade or pericarditis have been reported following cyst rupture or hemorrhage [1,3,18]. About 75–89% of patients with PCs are asymptomatic and are detected incidentally on imaging [3,5]. When symptoms occur, they are usually nonspecific and include cough, dyspnea, and/or palpitations [3]. These symptoms are due to the mass effect of the cyst and compression of surrounding structures. Large cysts have been reported to cause bronchial obstruction [19]. Rarely, a ruptured PC can cause cardiac tamponade [20]. There has been one case report of a ruptured PC presenting as acute pericarditis [18]. The differential diagnosis includes other paracardiac cystic lesions, such as thymic and bronchogenic cysts, which can be distinguished by their location and imaging features [3,21].

While approximately 75–89% of PCs cause no symptoms and are found incidentally, about 11–25% can become symptomatic or result in major complications, frequently requiring intervention [5,22]. Complications usually stem from the direct mechanical pressure on nearby structures, or from internal processes like infection, bleeding, or rupture.

### 4.1. Mechanical and Compressive Complications

The mass effect of the PC is the most frequent reason for complications.

- Cardiac Compression: Large cysts can exert pressure on adjacent cardiac chambers. This effect most often impacts the right atrium and right ventricle, potentially hindering diastolic filling and causing symptoms of right-sided heart failure or mimicking the hemodynamics of constrictive pericarditis [5,23]. Obstruction of the right ventricular outflow tract (RVOT) is also a well-recognized complication [24].
- Coronary Artery Compression: This is a rare but life-threatening complication where the cyst directly compresses a coronary artery, causing myocardial ischemia that can present as unstable angina or an acute myocardial infarction, requiring urgent intervention [25].
- Vascular and Airway Compression: Cysts can compress the superior vena cava, resulting in SVC syndrome, or the pulmonary veins, leading to breathlessness. Pressure on the trachea or a main bronchus can cause a chronic cough, shortness of breath, or repeated respiratory infections [24].
- Arrhythmias: Persistent mechanical irritation of the adjacent atrial wall is considered a possible cause of arrhythmias, with atrial fibrillation being the most common [26].

### 4.2. Inflammation, Infection, and Hemorrhage

- Infection and Abscess Formation: In exceedingly rare cases, chronic inflammation associated with a PC has been reported to cause either localized or widespread constrictive pericarditis, making the clinical situation more complex [27,28]. Cysts may become secondarily infected and develop into a pericardial abscess. This is a severe complication that presents with fever, chest pain, and elevated inflammatory markers, which requires urgent surgical drainage. Infected pericardial cysts with abscess formation are exceedingly rare complications, as most pericardial cysts remain benign and asymptomatic throughout their natural history [29]. When infection does occur, the microbiological etiology varies significantly based on geographic region and patient-specific risk factors. In developed countries, purulent pericardial infections are most commonly caused by Staphylococcus aureus and various Streptococcus species [30,31]. Organisms from normal skin flora, such as Propionibacterium acnes, may also be implicated, particularly in patients with predisposing factors including immunosuppression, chest wall trauma, or increased alcohol intake [30]. Geographic variation in etiology is substantial, with tuberculosis representing the dominant cause of pericardial infections in endemic regions. In much of the developing world, Mycobacterium tuberculosis accounts for approximately 70% of pericarditis cases in Africa and other low- and middle-income countries (LMICs), with mortality rates reaching 25% at 6 months in HIV-negative patients and 40% in those with HIV coinfection [32,33]. Hydatid disease caused by Echinococcus granulosus represents another important consideration in endemic regions, though cardiac involvement occurs in only 0.5–2% of echinococcosis cases [34]. Purulent pericarditis can develop as a complication when myocardial hydatid cysts rupture into the pericardial cavity with subsequent superinfection, or through fistula formation between infected hepatic hydatid cysts and the pericardium [35]. The literature on infected pericardial cysts specifically remains limited to case reports and small series, and further research is needed to better characterize the frequency, microbiology, and outcomes of this rare complication. On imaging, the infected pericardial cyst appears as a thick, enhancing wall and complex internal fluid [29].
- Hemorrhage: Spontaneous bleeding into the cyst can lead to a quick and painful expansion, triggering the sudden onset or worsening of compressive symptoms [36].
- Rupture and Cardiac Tamponade: Sudden rupture of a cyst into the pericardial space is the most feared, though rare, complication. This can rapidly lead to cardiac tamponade, a medical emergency that can cause hemodynamic collapse and sudden cardiac death [16].

## 5. Multimodality Imaging

While often first suspected on chest radiography or transthoracic echocardiography (TTE), a conclusive diagnosis and thorough assessment depend on advanced imaging like cardiac computed tomography (CT) and cardiac magnetic resonance (CMR). A comprehensive, multimodality imaging strategy is essential for precise characterization, assessing risk, and determining the appropriate course of management.

## 6. Echocardiography

**Figure 1:**
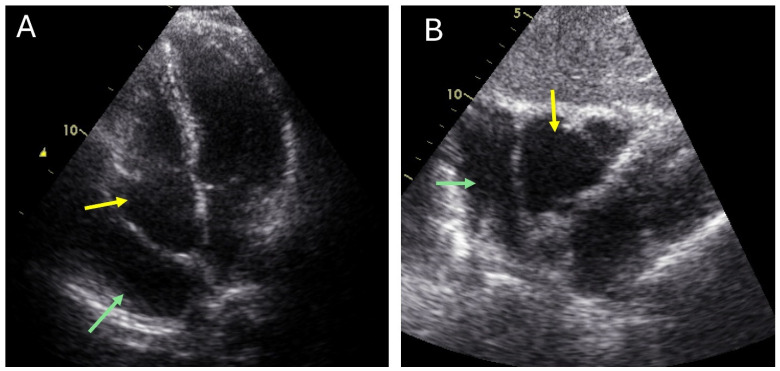
Transthoracic echocardiography of pericardial cyst (green arrow) adjacent to the right atrium (yellow arrow) in (**A**) Apical 4-chamber view and (**B**) Subcostal 4-chamber view.

As per the 2013 American Society of Echocardiography (ASE) guidelines and the 2024 international position statement from the American College of Cardiology (ACC), TTE serves as the primary imaging tool for diagnosing a PC [3,37]. On TTE, a PC typically appears as a thin-walled, anechoic (echo-free) space located next to a heart chamber, most commonly the right atrium [3] (Figure 1). It is vital to use color Doppler to verify the absence of blood flow within the structure, which helps distinguish it from vascular anomalies [3,37]. However, TTE has a limited sensitivity, particularly for cysts located at the right heart border, where acoustic windows can be challenging [23]. A study by Alkharabsheh et al. found that it detected only 34 of 103 cases [5]. The sensitivity was noted to be higher for left-sided cysts (60%) [38]. Employing specialized off-axis and superiorly angled subcostal views can improve visualization [23].

Beyond cyst detection, TTE provides a comprehensive hemodynamic assessment that is critical when complications arise. TTE is used to identify the presence of pericardial effusion, sizing of the effusion as well as assess for cardiac tamponade. It further allows evaluation of chamber compression, respiratory variation in ventricular filling, and other tamponade physiology—particularly important since PC rupture with hemorrhage can potentially cause cardiac tamponade [3,37]. For constrictive physiology assessment, TTE can evaluate ventricular interdependence, respiratory variation in mitral and tricuspid inflow velocities, and tissue Doppler imaging findings consistent with constriction, like annulus reversus and annulus paradoxus. This is relevant when pericardial disease extends beyond simple cysts. Standard TTE evaluation of cardiac chambers and valves proceeds normally in the presence of PCs, where it can help assess left and right ventricular size and systolic function, diastolic parameters, and valvular structure and function. Additionally, large cysts causing extrinsic compression may affect chamber size or function, which TTE can detect [3]. There is no specific role of stress echocardiography in the evaluation of PCs. Stress echocardiography is designed to assess for inducible myocardial ischemia and coronary artery disease through exercise or pharmacologic stress, which is not relevant to the diagnosis or management of pericardial cysts—benign structural lesions that do not involve the coronary circulation or myocardial perfusion. Transesophageal echocardiography provides better spatial detail and is especially valuable for examining cysts in atypical posterior locations or for evaluating hemodynamic impact on adjacent structures, but is seldom needed for clinical evaluation [38].

## 7. Cardiac Computed Tomography

**Figure 2:**
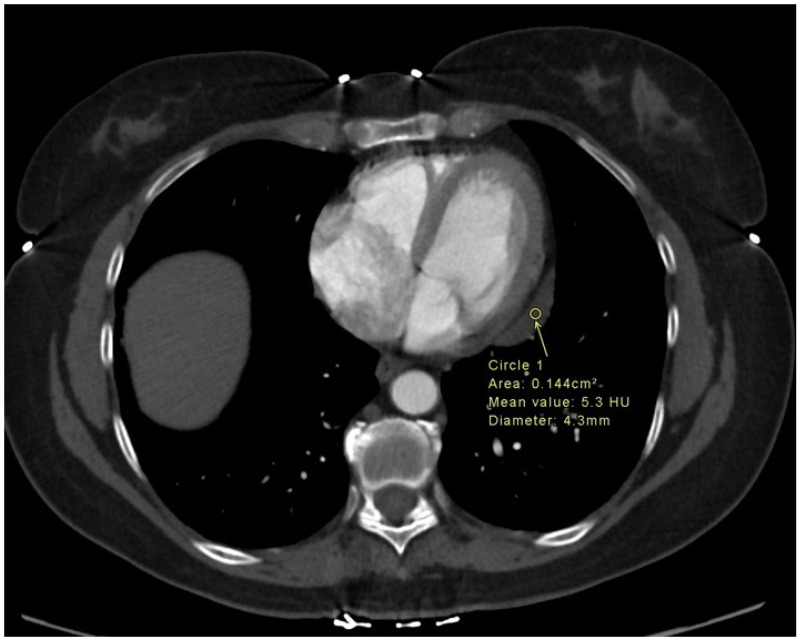
Axial computed tomography chest images depicting an incidentally detected well-defined hypoattenuating cystic lesion (yellow arrow) along the basal-mid lateral left ventricular wall consistent with a pericardial cyst.

CT is excellent for determining the precise anatomical position of the cyst and clarifying its spatial relationship with both cardiac and non-cardiac structures. The characteristic CT appearance is a well-defined, thin-walled, non-enhancing, single-chambered oval mass that contains fluid with attenuation of 0–40 Hounsfield units (HU) values similar to water [37]. Unlike a pericardial diverticulum, a PC does not have an open connection to the pericardial sac [3]. For complex cysts containing proteinaceous or hemorrhagic material, the attenuation values may be higher, which can mimic a solid mass. Complex cysts with high protein content show attenuation > 10 HU [3]. Pericardial effusions are characterized by their composition: transudative effusions measure 0–20 HU, exudative effusions 20–50 HU, and hemorrhagic effusions > 50–60 HU [3,37]. Blood typically demonstrates attenuation of 40–88 HU when non-sedimented [39]. Figure 2 depicts the CT finding of PC.

Both CT and TTE provide complementary information for defining pericardial cyst location and guiding drainage when indicated [3,40]. TTE serves as the first-line modality, identifying cysts as echo-free spaces adjacent to the cardiac border, though visualization depends on acoustic windows [37]. CT provides superior anatomic detail, delineating cyst size, precise location, and involvement of surrounding structures, which is particularly valuable for guiding percutaneous drainage in challenging cases [3,37,41]. CT can identify loculated effusions and guide CT-guided pericardiocentesis when echocardiographic windows are suboptimal [37,40].

CT comprehensively evaluates multiple life-threatening causes of chest pain in a single examination. For coronary artery disease, cardiac CT angiography assesses coronary anatomy and stenosis severity. CT pulmonary angiography definitively diagnoses pulmonary embolism, while CT aortography evaluates for aortic dissection [42,43]. CT also identifies lung pathology including pneumonia, pneumothorax, and malignancy. Pericardial calcifications are readily detected on CT, appearing as high-attenuation deposits that may suggest constrictive pericarditis when present [41]. Triple rule-out CT protocols can simultaneously evaluate coronary arteries, pulmonary arteries, and aorta, though dedicated protocols are preferred when the differential diagnosis can be narrowed [43].

## 8. Cardiac Magnetic Resonance

CMR is widely regarded as an excellent modality for non-invasive tissue characterization of PCs and for distinguishing them from other masses in the mediastinum, like pericardial diverticulum, without the use of ionizing radiation [37]. Using standard imaging sequences, a simple PC shows uniform low signal on T1-weighted images and a distinct high signal on T2-weighted images, which is characteristic of simple fluid [23]. After administering gadolinium-based contrast, a simple cyst will remain unenhanced, confirming its avascular nature [44]. Recent advancements have further solidified the diagnostic role of CMR.

- Parametric Mapping: Quantitative T1 and T2 mapping allows for precise characterization of the fluid. Higher T1 and T2 values can suggest complex contents, such as hemorrhage or a high protein concentration [45].
- Diffusion-Weighted Imaging (DWI): DWI, along with Apparent Diffusion Coefficient (ADC) mapping, helps differentiate benign cysts from malignant or infected masses. Simple PCs demonstrate unrestricted diffusion with high ADC values, while malignant tumors and abscesses usually show restricted diffusion and lower ADC values [46].
- Late Gadolinium Enhancement (LGE): While simple cysts are non-enhancing, LGE imaging is critical when infection or inflammation is suspected. Enhancement of the cyst wall or adjacent pericardium points indicates an active inflammatory process, such as pericarditis or a complicated (e.g., infected) collection [3,37].

CMR’s comprehensive pericardial assessment extends beyond cyst detection to provide detailed tissue characterization that distinguishes active inflammation from chronic fibrotic changes [41,47]. The integration of multiple CMR sequences within a single examination allows simultaneous evaluation of pericardial anatomy, inflammation, effusion characteristics, and hemodynamic consequences—information particularly valuable when PCs occur in the context of broader pericardial disease [47].

Pericardial late gadolinium enhancement (LGE) demonstrates very high sensitivity (94–100%) and specificity for pericardial inflammation, reflecting increased pericardial vascularity and providing incremental diagnostic information beyond conventional clinical criteria for pericarditis [48]. The combined evaluation of LGE with T2-STIR sequences enables disease staging: prominent LGE with increased T2 signal indicates acute inflammation, increased LGE with normal T2 signal suggests subacute inflammation with edema resolution, and LGE without elevated T2 signal represents chronic inflammation [30].

Black-blood spin-echo imaging provides optimal assessment of pericardial thickness, with measurements > 4 mm considered abnormal, though some patients with constrictive pericarditis may have normal pericardial thickness [41,49]. Steady-state free precession cine sequences assess pericardial effusion presence and quantity while simultaneously identifying morphologic features of constriction, including conical and tubular ventricular deformities, ventricular tethering, and inferior vena cava enlargement [41,50]. Free-breathing cine imaging demonstrates respirophasic septal shift representing ventricular interdependence, the hemodynamic hallmark of constrictive physiology, with relative septal excursion being a specific finding for constriction [50].

**Figure 3:**
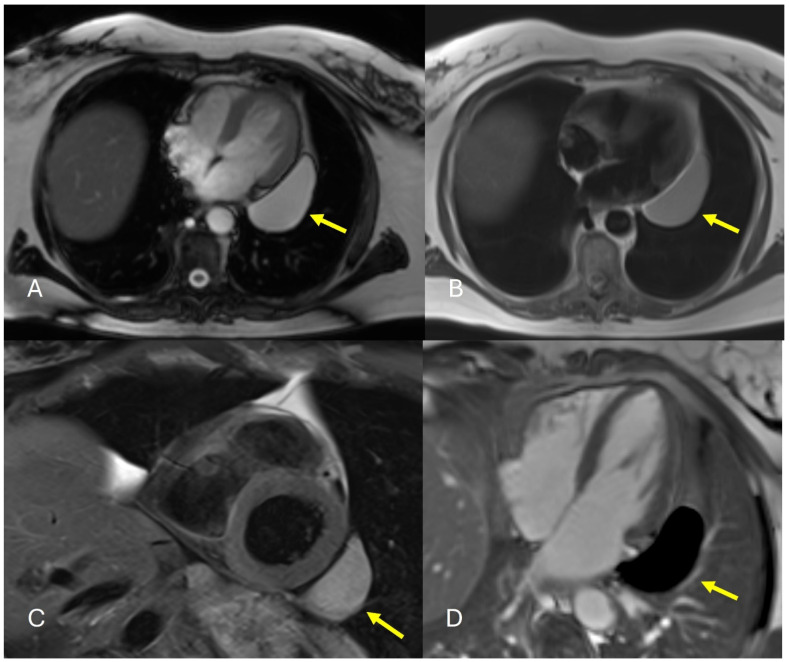
(**A**–**D**): Cardiac Magnetic Resonance imaging on the same patient (performed a few years later) depicting interval increase in size of the well-defined lesion along the left lateral heart border (yellow arrow), which appears hyperintense on axial white blood (**A**), axial black blood (**B**), T2 fat sat short axis (**C**) images with no evidence of internal or peripheral enhancement on post contrast images (**D**), consistent with pericardial cyst.

CMR also characterizes pericardial effusion composition based on signal characteristics across different sequences, distinguishing simple transudates from complex exudates or hemorrhagic effusions [41]. However, T2-STIR sequences can be challenging to interpret when pericardial effusion is present, as the high water content of effusions can make distinguishing concomitant pericardial edema difficult [41,49]. Additionally, CMR requires a stable heart rhythm and adequate breath-holding for optimal image quality, and gadolinium administration is contraindicated in advanced renal dysfunction [30]. Figure 3 demonstrates the CMR finding of PC. The Supplemental Video Clip S1 with cine short axis and 4 chamber view demonstrates a pericardial cyst along the lateral left ventricular wall. The multimodality imaging characteristics are summarized in Table 1.

## 9. Nuclear Imaging

Although it is not a routine diagnostic method, ^18^F-Fluorodeoxyglucose Positron Emission Tomography (^18^F-FDG PET/CT) is an important problem-solving technique. It is highly sensitive for detecting metabolic activity associated with inflammation, infection, or malignancy, often showing high metabolic activity on ^18^F-FDG PET/CT. This has been reported even in pediatric cases, highlighting the need for vigilance [29]. When an infected PC or one secondary to a malignancy is suspected, significant FDG uptake in the cyst wall can validate the diagnosis, help direct treatment, and monitor the response to therapy [51].

## 10. Imaging in Special Populations

PCs can be diagnosed prenatally via ultrasound, usually around 25 weeks of gestation. For these cases, repeated sonography is advised to monitor for growth and potential compression of fetal anatomy [23]. Follow-up evaluation with echocardiography and CMR is required after birth. In children, especially infants, these cysts can cause severe compressive symptoms or become infected, which makes prompt and precise imaging essential for diagnosis and surgical preparation [29].

Prognostically, PCs are benign manifestations that occur in the pericardium and have a good prognosis. Most cysts are found by accident on imaging while looking for something else, and remain asymptomatic throughout life [4,16]. It has been suggested that the majority of PCs have natural stability with a minimal risk of enlargement or progression, and many remain unchanged over long-term follow-up [23,36]. Spontaneous regression has been observed in some cases, possibly due to rupture of the cyst into the pleura or pericardium, leading to its resolution without any clinical consequences [36].

## 11. Surveillance and Monitoring

The diagnostic process for a suspected PC starts with TTE as the initial imaging choice [3,37]. If a cystic formation is seen or if clinical suspicion is high, even with an inconclusive TTE, the next step is advanced cross-sectional imaging to confirm the diagnosis, precisely delineate anatomy, and characterize the tissue. CMR is typically favored over CT for this, offering better soft-tissue detail and avoiding ionizing radiation, which makes it perfect for differentiating a simple cyst from a complex or solid mass [37].

For an asymptomatic patient with a definitively diagnosed simple PC, guidelines advocate for a conservative “watch-and-wait” approach. The ASE recommends serial imaging every 1 to 2 years to monitor for stability, preferably with CMR [3]. The emergence of new symptoms or any sign of cyst growth on subsequent imaging should lead to a reassessment of the management plan [5].

**Figure 4:**
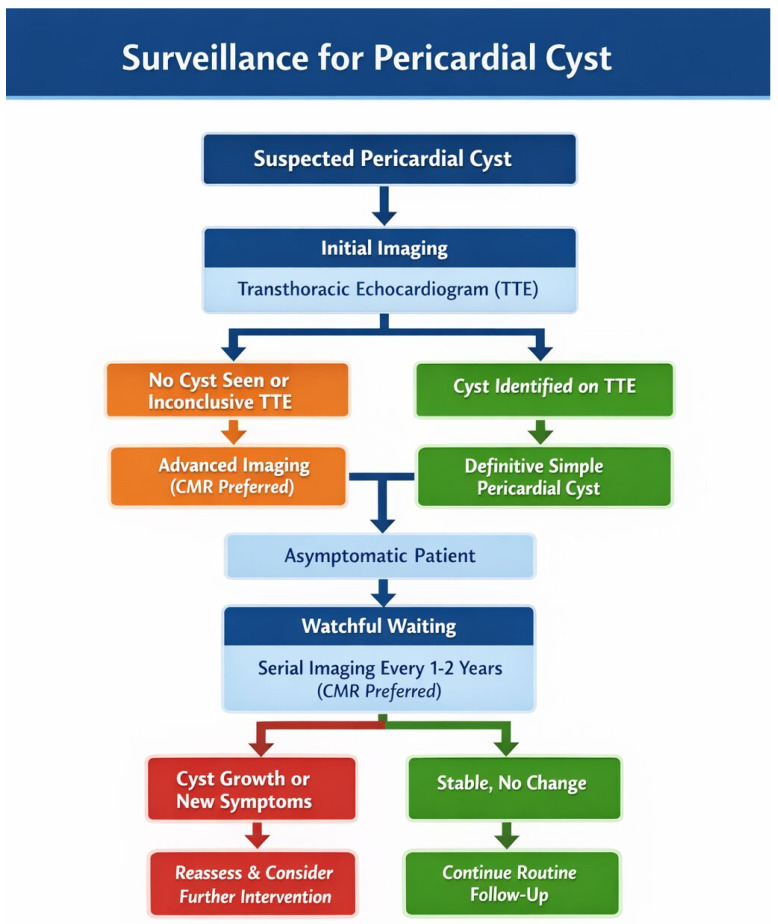
Flowchart for surveillance of pericardial cysts.

Transthoracic echocardiography offers the advantage of being widely available, radiation-free, and convenient for serial surveillance, but provides limited anatomic detail and may not clearly visualize cysts, depending on location [37]. CT provides superior spatial resolution and anatomic delineation, but it involves cumulative radiation exposure with repeated imaging [52]. CMR combines excellent tissue characterization with the absence of radiation, making it ideal for long-term follow-up, particularly in symptomatic patients requiring serial assessment [37,52]. Figure 4 depicts the surveillance algorithm of pericardial cysts.

## 12. Management

Long-term results are good, no matter the treatment or management. In asymptomatic patients, conservative observation with serial imaging is safe, and most cysts remain stable in size [23].

In the setting of pericarditis, the medical management follows the standard pericarditis treatment protocols, as pericardial cysts themselves are typically benign and do not require specific anti-inflammatory therapy unless complicated by pericardial inflammation. The ACC recommends first-line dual therapy with colchicine (0.6 mg twice daily for patients > 70 kg, or 0.6 mg once daily for ≤70 kg) for 3 months following first flare or ≥6 months following recurrence, combined with high-dose NSAIDs (ibuprofen 600–800 mg three times daily or aspirin 650–1000 mg three times daily) that are tapered after symptom resolution and normalization of inflammatory markers [40]. Gastroprotection with proton pump inhibitors is recommended with NSAIDs [31,40]. For patients with an inflammatory phenotype (elevated CRP >1 mg/dL and/or LGE on CMR) who fail first-line therapy or have contraindications, anti-IL-1 agents such as rilonacept or anakinra are now preferred over corticosteroids [40]. Corticosteroids (prednisone 0.2–0.5 mg/kg/day) are reserved for refractory cases or those without an inflammatory phenotype, given their side effect profile [40]. Exercise restriction (maximal heart rate <100 beats/min) for ≥1 month until clinical remission is important, as increased heart rate can trigger pericardial inflammation [40]. If the pericardial cyst has ruptured, causing hemorrhagic pericarditis or tamponade, treatment focuses on managing the acute pericardial inflammation while addressing the mechanical complication through drainage or surgical intervention as needed [3,37].

There is limited data on percutaneous sclerotherapy and it comes from small case series. Ethanol sclerosis has been reported as successful in individual cases, with one series describing complete symptom relief and no recurrence at 6-month follow-up in two patients treated with alcohol ablation under pericardioscopic control [53]. Another case report documented successful treatment with percutaneous aspiration and ethanol injection without recurrence at 6 months [54]. However, these represent anecdotal evidence rather than systematic comparative data. The ASE guidelines indicate that indications for resection or drainage include symptoms, large size, and potential for rupture or malignancy, but do not specify exact size cutoffs [3]. The literature demonstrates successful conservative management of even very large cysts, including one of the largest reported cases in a 102-year-old patient, suggesting that size alone does not mandate intervention in asymptomatic patients [1]. Natural history data show that approximately one-third of pericardial cysts decrease in size over time (mean 25%), while only 17% show modest enlargement (mean 13%), with most remaining asymptomatic during follow-up [5]. Current practice emphasizes conservative management with observation or serial imaging (CT or CMR every 1–2 years) for asymptomatic patients regardless of size, with intervention reserved for symptomatic cases or diagnostic uncertainty [3,37].

For patients with symptoms or an enlarged cyst, surgical excision or percutaneous drainage is usually curative and causes little morbidity [55]. Video-assisted thoracoscopic surgery (VATS) is another minimally invasive procedure that improves outcomes by reducing the recovery period, reducing pain, and almost eliminating recurrence [55,56,57]. According to a single-center retrospective study by Zhang et al., surgical management can result in complete relief of symptoms with no recurrence during follow-up [55]. Robotic-assisted thoracic surgery (RATS) has emerged as a safe and effective minimally invasive approach for pericardial cyst resection, offering several advantages over traditional VATS and open thoracotomy [58,59]. The robotic platform provides enhanced three-dimensional visualization with high-definition magnification, improved instrument dexterity with wristed articulation, and greater precision in dissection, which is particularly beneficial when managing cysts adherent to critical mediastinal structures or located in challenging anatomical positions such as the cardiophrenic angles [59,60,61,62]. From an ergonomic perspective, RATS offers superior surgeon comfort through console-based operation with tremor filtration and intuitive instrument control, eliminating the physical strain associated with prolonged VATS procedures [61,62]. Comparative studies of mediastinal tumor resection have demonstrated that RATS is associated with reduced intraoperative bleeding, lower conversion rates to open thoracotomy (4.9% vs. 14.7%), fewer postoperative complications, shorter chest tube duration, and decreased length of hospital stay compared to VATS, even for lesions exceeding 4 cm in diameter [60,63]. Single-site robotic approaches have also been successfully employed for pericardial cysts, with median operative times of approximately 105 min, minimal postoperative pain (median peak score of 3), and short hospital stays (median 4 days) [59]. While RATS demonstrates superior perioperative outcomes and may be cost-effective when considering reduced length of stay and complications, it is associated with higher upfront hospitalization costs compared to VATS, with incremental cost-effectiveness ratios ranging from $113,389 to $180,755 per quality-adjusted life-year depending on institutional factors and surgical volume [63,64,65,66]. Complete excision remains essential regardless of approach to prevent recurrence, and the robotic platform facilitates thorough resection while minimizing morbidity, making it an increasingly preferred technique for symptomatic pericardial cysts requiring surgical intervention [60,62,67].

After surgery or a very tiny incision, it is very rare for it to come back. The long-term outcome is excellent. The follow-up surgery is usually done after 6 to 12 months to check if there is residual or recurrent cystic tissue [55,57]. After this time, further imaging is usually no longer necessary unless new symptoms occur. In general, both conservative and surgical management have excellent outcomes, and patients will live a normal life expectancy without significant cardiac sequelae [4,23,55].

Empiric antibiotic coverage for an infected mediastinal cyst before culture results should follow guidelines for empyema and surgical infections, with regimen selection based on whether the infection is community-acquired or hospital-acquired/postprocedural. For community-acquired infections, the American Association for Thoracic Surgery recommends a parenteral second- or third-generation cephalosporin (such as ceftriaxone) with metronidazole, or a parenteral aminopenicillin with β-lactamase inhibitor (such as ampicillin-sulbactam) [68]. Ampicillin-sulbactam provides inherent anaerobic coverage, while ceftriaxone requires the addition of metronidazole or clindamycin for anaerobic activity. Anaerobic coverage is essential given the frequency of anaerobic organisms in mediastinal infections and the inconsistent success of culturing these organisms [68].

For hospital-acquired or postprocedural infections, empiric coverage must include antibiotics active against methicillin-resistant Staphylococcus aureus (MRSA) and Pseudomonas aeruginosa, such as vancomycin plus cefepime and metronidazole, or vancomycin plus piperacillin-tazobactam (dosed adequately for Pseudomonas). Vancomycin and meropenem may be indicated if extended-spectrum β-lactamase-producing organisms are suspected. Aminoglycosides should be avoided as they are inactivated in purulent fluid and are not recommended for empyema or mediastinal infections. Empiric therapy should be tailored to local antimicrobial resistance patterns and institutional stewardship policies, then narrowed based on culture results when available [68].

Management of pyogenic versus hydatid mediastinal cysts differs fundamentally in surgical approach and perioperative considerations. Pyogenic cysts require prompt surgical drainage with broad-spectrum antibiotics and source control [69,70]. One case report documented successful treatment of an infected mediastinal cyst with teicoplanin plus imipenem/cilastatin along with ultrasound-guided transcutaneous catheterization drainage [70].

In contrast, hydatid cysts require meticulous surgical technique to avoid catastrophic complications from cyst rupture, including anaphylactic shock, secondary peritoneal or pleural seeding, and dissemination [71,72,73]. Rupture of hydatid cysts during surgery can lead to severe complications including mediastinal suppuration and empyema [72,74]. Preoperative antihelminthic therapy with albendazole (preferred over mebendazole) is essential to decrease cyst pressure, prevent secondary seeding, and mitigate anaphylaxis risk during percutaneous drainage or surgery [71]. Medical therapy should be initiated before surgical or percutaneous procedures and continued for 1–6 months afterward, with cyclical regimens no longer recommended given the parasitostatic activity and safety profile of continuous therapy [75].

Surgical management of hydatid cysts demands specialized precautions: meticulous control of cyst content spillage, use of scolicidal agents, and complete excision without delay to avoid compression of vital structures [71,73]. Surgery remains the treatment of choice for mediastinal hydatid cysts, particularly those at risk of rupture, and should be performed urgently when rupture is suspected [72,73]. Hydatid disease management requires interdisciplinary collaboration between infectious disease specialists and surgeons, with treatment individualized based on cyst stage (WHO classification), size, location, and patient-specific factors [71]. The management of PCs is summarized in Table 2.

## 13. Limitations

This review has several limitations. First, as a narrative review, the literature search and study selection were nonsystematic, which introduces the potential for selection bias and systematic error. Second, no formal assessment of study heterogeneity or risk of bias was performed. This reflects the nature of the available evidence rather than an oversight in methodology. Pericardial cysts are rare entities, and the existing literature is predominantly composed of case reports, small case series, and observational studies with substantial clinical and methodological heterogeneity. As a result, quantitative synthesis, meta-analysis, or formal heterogeneity assessment was not feasible.

Conclusions regarding diagnostic strategies and management approaches are based largely on descriptive data, expert opinion, and consensus recommendations rather than high-level evidence. Diagnostic challenges persist, as pericardial cysts may be confused with localized pericardial effusion, tumor, ventricular aneurysm, or cardiomegaly without further work-up, and differentiation from other mediastinal cysts (bronchogenic, thymic, enteric) requires advanced imaging with CMR or CT. Additionally, the literature lacks robust data on long-term outcomes, complication rates, and quality-of-life measures following different treatment approaches, limiting the ability to provide evidence-based counseling to patients regarding optimal management strategies. Despite these limitations, this review provides a comprehensive and clinically relevant synthesis of current knowledge regarding the evaluation and management of pericardial cysts.

## 14. Conclusions

In summary, PCs are rare, benign pericardial lesions that are most often discovered incidentally and typically follow a stable or slow-growth course, resulting in an excellent long-term prognosis. Most remain asymptomatic throughout life, and complications such as infection, rupture, hemorrhage, or cardiac tamponade are exceedingly uncommon. Management should be individualized based on symptoms and cyst characteristics. Advances in imaging and minimally invasive surgical techniques have further improved the safety and precision of PC management by providing better characterization of cyst size, location, and impact on surrounding structures, enabling more informed clinical decision-making. Timely recognition and appropriate management of symptomatic or complicated cysts help prevent adverse events while preserving excellent long-term outcomes.

## Figures and Tables

**Table 1:** Summary of multi-modality imaging in pericardial cyst.

Modality	Indications	Advantages	Disadvantages	Surveillance Recommendations
**Transthoracic Echocardiography (TTE)**	First-line imaging for incidental detection Hemodynamic assessment (effusion, tamponade) Assessment of chamber size/function and valves	Widely available and convenient No radiation exposure Real-time hemodynamic evaluation Can guide pericardiocentesis	Limited anatomic detail Location-dependent visualization May not clearly distinguish cyst from artifact or localized pericardial effusion Cannot reliably differentiate cyst from diverticulum	Reasonable for serial follow-up in asymptomatic patients Optimal frequency not well established
**Computed Tomography (CT)**	Confirmation when TTE is inconclusive Defines cyst size, location, and extent Guides percutaneous drainage Evaluates alternative causes of chest pain (coronary artery disease, pulmonary embolism, dissection)	Superior spatial resolution and anatomic detail Detects pericardial calcifications Attenuation characterization (simple: 0–40 Hounsfield units (HU); hemorrhagic: >50–60 HU) Evaluates surrounding structures	Radiation exposure with serial imaging Contrast limitations (renal dysfunction, allergy) Inferior tissue characterization vs. CMR	Useful to confirm and monitor cyst size Surveillance every 1–2 years in symptomatic patients Limited role in asymptomatic patients
**Cardiac Magnetic Resonance (CMR)**	Tissue characterization when diagnosis is uncertain Differentiates simple vs. complex/hemorrhagic cysts Distinguishes cyst from diverticulum or mediastinal masses Serial imaging in symptomatic patients	Excellent tissue characterization without radiation Simple cysts: low–intermediate T1, high T2 Hemorrhagic/complex: high T1 and T2 No gadolinium enhancement in simple cysts Ideal for long-term surveillance	Limited availability Longer acquisition time Requires rhythm stability and breath-holding Gadolinium is contraindicated in severe renal dysfunction Higher cost	Preferred modality for serial follow-up in symptomatic patients Surveillance every 1–2 years Observation is appropriate in asymptomatic patients

**Table 2:** Management of Pericardial Cyst.

Clinical Scenario	Management Strategy	Key Details/Notes
Asymptomatic pericardial cyst (no inflammation)	Conservative management	Cysts are typically benign; no anti-inflammatory therapy is required
Pericarditis associated with a pericardial cyst	Standard pericarditis therapy	Treat as per guideline-based pericarditis; the cyst itself does not require specific therapy unless complicated
All pericarditis cases	Exercise restriction	Max HR <100 bpm for ≥1 month until clinical remission
Ruptured cyst with hemorrhagic pericarditis or tamponade	Acute management + mechanical intervention	Treat inflammation and perform pericardial drainage or surgery as needed
Symptomatic or enlarging pericardial cyst	Surgical excision or percutaneous drainage	Usually curative with low morbidity
Preferred surgical approach	Video-assisted thoracoscopic surgery (VATS)	Minimally invasive; less pain, faster recovery, near-zero recurrence
Post-surgical follow-up	Imaging surveillance	Follow-up imaging at 6–12 months to assess residual/recurrent cyst
Long-term management	No routine imaging if asymptomatic	Further imaging only if new symptoms develop
Overall prognosis	Excellent	Normal life expectancy with minimal risk of long-term cardiac sequelae

## Data Availability

This article is a review of previously published studies. No new datasets were generated or analyzed during the current study. All data discussed are available in the cited references.

## Supplementary Materials

The following supporting information can be downloaded at: https://www.mdpi.com/article/10.3390/jcm15041585/s1, Video S1: Cine images in short axis and 4-chamber images depicting the pericardial cyst along the lateral left ventricular wall, showing no appreciable mass effect. No evidence of constrictive physiology.
